# DDIG-in: discriminating between disease-associated and neutral non-frameshifting micro-indels

**DOI:** 10.1186/gb-2013-14-3-r23

**Published:** 2013-03-13

**Authors:** Huiying Zhao, Yuedong Yang, Hai Lin, Xinjun Zhang, Matthew Mort, David N Cooper, Yunlong Liu, Yaoqi Zhou

**Affiliations:** 1 School of Informatics, Indiana University Purdue University Indianapolis, 719 Indiana Ave., WK Bldg Suite 319, Indiana 46202, USA; 2 Center for Computational Biology and Bioinformatics, Indiana University School of Medicine, 410 West 10th Street, HITS Bldg Suite 5000, Indianapolis, Indiana 46202, USA; 3 Institute of Medical Genetics, Cardiff University, Heath Park, Cardiff CF14 4XN, UK; 4 Department of Medical and Molecular Genetics, Indiana University School of Medicine, 975 West Walnut Street, MRL Bldg IB130, Indianapolis, Indiana 46202, USA

## Abstract

Micro-indels (insertions or deletions shorter than 21 bps) constitute the second most frequent class of human gene mutation after single nucleotide variants. Despite the relative abundance of non-frameshifting indels, their damaging effect on protein structure and function has gone largely unstudied. We have developed a support vector machine-based method named DDIG-in (Detecting disease-causing genetic variations due to indels) to prioritize non-frameshifting indels by comparing disease-associated mutations with putatively neutral mutations from the 1,000 Genomes Project. The final model gives good discrimination for indels and is robust against annotation errors. A webserver implementing DDIG-in is available at http://sparks-lab.org/ddig.

## Background

The largest class of human gene mutation is the single nucleotide variant (SNV) which comprises approximately 67% of known pathological mutations [1]. This is followed by microinsertions and microdeletions (micro-indels of ≤20 bp) which together comprise approximately 22% of known pathological mutations [2]. In addition, with the broad implementation of next generation sequencing technology in genetic studies, several million polymorphic micro-indels have been identified and analyzed in the human genome [3-7]. Many more genetic variants, including micro-indels, are currently being discovered at an unprecedented rate. Obviously, it is impractical to examine the impact of each variant on biological function individually. Hence, there is a critical need for effective bioinformatics tools that are capable of distinguishing potentially disease-causing variants from those that are functionally neutral.

Most available tools for prioritizing genetic variants are however limited to non-synonymous SNVs. Examples are SIFT [8], POLYPHEN [9], and MutPred [10] (for recent reviews, see [11-15]). These tools are not however applicable to indels because indels change the number of nucleotides in the gene and hence may be expected to have a much greater impact on molecular function than single nucleotide substitutions. There are two main types of indel within exons: frameshifting (FS) and non-frameshifting (NFS). NFS-indels insert/delete multiples of three nucleotides leading to the addition or removal of specific amino-acid residues at the indel site. FS-indels, on the other hand, insert/delete a discrete number of nucleotides that are indivisible by three and therefore alter the entire reading frame resulting in either a completely different amino-acid sequence C-terminal to the indel site, or premature termination of translation. Two bioinformatics methods were recently designed to discriminate between functional and non-functional FS-indels [16,17] and nonsense mutations (premature stop codons) [16]. However, to our knowledge, there is no technique available that is capable of analyzing NFS-indels. Methods for interrogating FS-indels would not be applicable to NFS-indels because FS-indels modify the entire amino-acid sequence C-terminal to the indel site (unless a second indel were to exist), whereas NFS-indels simply alter the amino-acid sequence at the indel site. Such a technique for NFS-indel prioritization is urgently required because NFS-indels constitute a significant fraction of all exonic indels (theoretically, it is about one-third). In practice, we found that only 26% of 9,327 exonic micro-indels are NFS indels in the 1,000 Genomes Project data [18].

In this paper, we have developed a method that we have termed *DDIG-in *(Detecting DIsease-causing Genetic variants due to microinsertions/microdeletions) to prioritize NFS-indels by comparing disease-causing indels from the Human Gene Mutation Database (HGMD) [1] with putatively neutral NFS-indels from the 1,000 Genomes Project [18], respectively. We developed and examined a total of 58 sequence- and structure-based features of indel sites and found that the feature based on predicted unstructured regions by disorder predictor SPINE-D [19] was the most discriminating one. This feature can, on its own, achieve a value of 0.56 for the Matthews Correlation Coefficient (MCC), and 0.82 for the area under the receiver-operating characteristic (ROC) curve (AUC). We developed two separate support vector machines (SVM) methods for NFS-microdeletions and NFS-microinsertions that were 10-fold cross-validated and independently tested on microinsertions and microdeletions, respectively. A similar level of accuracy between independent testing and 10-fold cross-validation indicates not only the robustness of our training procedure but also a similar deleterious impact of NFS microdeletions and microinsertions. Of the 58 features tested (listed in Table 1), nine features were selected by maximizing the discriminatory roles for detecting disease-causing NFS microinsertions and microdeletions in a non-redundant dataset of micro-indels. Our *DDIG-in *method received further confirmation from the observation that NFS-indel variants with higher predicted disease-causing probabilities were characterized by lower average minor allele frequencies in the general population (based on data from the 1,000 Genomes Project).

**Table 1:** List of all features considered.

Features	Description
*Nucleotide level *	
Microdeletion/microinsertion positions (2)	Distances to nearest 5' and 3' splicing positions
DNA conservation scores (3)	Maximum, minimum, average
*Protein level*	
Evolution feature (30)	Maximum, minimum, average values (7 transition probabilities between match (M), microdeletion (D), and microinsertion (I) (MM, MI, MD, IM, II, DM, DD), 3 effective numbers of match/microinsertion/microdeletion)
Length (4)	Protein length, Microdeletion/microinsertion length, Distances to terminals
ΔS (1)	The indel-induced change to the HMM match score
Disorder score (3)	Maximum, minimum, average
Secondary structure (12)	Maximum, minimum, average probability (C, H, E), Predicted secondary structure (C, H, E)
Accessible surface area (3)	Maximum, minimum, average

## Results

### Single feature performance

**Figure 1:**
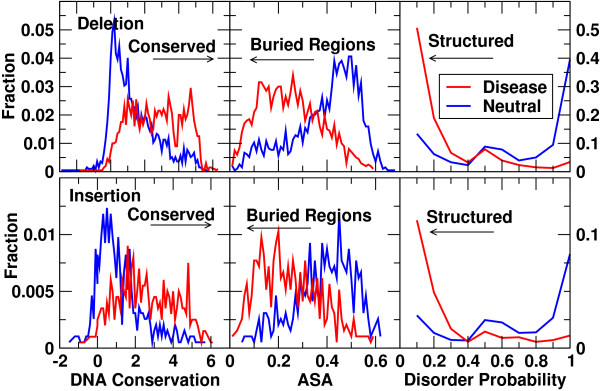
**Distributions of the average DNA conservation score from phyloP (phylogenetic *P *values) (left), the average solvent accessible surface area (ASA, middle), and the average disorder probability (right) of disease-causing (red) and neutral (blue) indels (microdeletions (top panel) and microinsertions (bottom panel))**.

We first examined the ability of a single feature to discriminate between disease-causing and neutral NFS-indels. Table 2 compares the top five performing features for microdeletions and microinsertions, separately, based on a half-window size of 2 (n_window_=2). A more complete list can be found in Additional file 1, Tables S1 (deletion) and S2 (insertion). Similar results were obtained with different window sizes (see Discussion). The results indicated that the top two performing features for microinsertions and microdeletions were both the same (disorder and solvent accessible surface area). This was followed by DNA conservation or effective number of homologous sequences aligned to residues instead of gaps. Both features represent evolutionary conservation scores but at the nucleotide and amino-acid residue levels, respectively. The effective number of homologous sequences aligned to amino-acid residues can be regarded as the conservation of amino-acid sequence position (not aligned to microdeletion or microinsertion regions). The fifth most discriminative feature was predicted sheet probabilities for microdeletions and transition probabilities between microinsertion and match for microinsertions. Inspection of Table 2 reveals that a single disorder feature alone can achieve an MCC value of 0.56 and an AUC of 0.82. At this MCC value, it has 74% precision and 85% recall (or sensitivity). Figure 1 depicts the distributions of DNA conservation score, disorder probability, and solvent accessible surface area (ASA) for the disease-causing and putatively neutral microdeletions (Figure 1 top) and microinsertions (Figure 1 bottom), respectively. It is clear that the disease-causing NFS-indels occur more frequently within regions characterized by a greater degree of evolutionary conservation at the nucleotide level, lower disorder probability (structural regions), and lower ASA (buried core regions). The results summarized in Table 2 and Figure 1 support the view that disruption of protein structure (and hence protein function) is the single most important reason why the NFS-indels are deleterious from the various features examined. Similar top-ranked features for microdeletions and microinsertions suggest that a single predictive method may be developed for microinsertions and microdeletions combined.

**Table 2:** Top five performing features for microdeletion and microinsertion discrimination.

Features	MCC^a^	AUC^b^	Precision	Recall
*Deletion*				

Disorder (Min, Ave, Max)	0.558, 0.557, 0.551	0.824, 0.825, 0.818	74%, 74%, 73%	85%, 85%, 84%
ASA^c ^(Min, Ave, Max)	0.542, 0.47, 0.302	0.81, 0.781, 0.659	73%, 71%, 68%	88%, 81%, 57%
DNA conservation (Max, Ave, Min)	0.468, 0.367, 0.144	0.781, 0.742, 0,561	68%, 72%, 66%	79%, 71%, 23%
Neff^d ^(Min, Ave, Max)	0.449, 0.439, 0.43	0.735, 0.749, 0.729	68%, 66%, 67%	85%, 87%, 85%
Probability of sheet (Max, Min Ave)	0.32, 0.305, 0.284	0.678, 0.658, 0.632	69%, 69%, 64%	60%, 53%, 51%

*Insertion *				

Disorder (Min, Max, Ave)	0.556, 0.546, 0.545	0.813, 0.816, 0.80	78%, 80%, 79%	75%, 74%, 75%
ASA^c ^(Min, Ave, Max)	0.501, 0.454, 0.317	0.80, 0.78, 0.670	71%, 78%, 71%	85%, 65%, 52%
Neff ^d ^(Min, Ave, Max)	0.467, 0.455, 0.438	0.751, 0.747, 0.742	68%, 68%, 67%	86%, 85%, 84%
DNA conservation (Max, Ave, Min)	0.453, 0.422, 0.234	0.758, 0.752, 0.597	72%, 74%, 76%	75%, 65%, 27%
Transition probability of microinsertion to match (Min)	0.372	0.708	72%	62%

Note: Max, min, and ave are arranged in the order of MCC values.

ASA, Solvent accessible surface area; AUC, Area under the curve; MCC, Matthews correlation coefficient; Neff, the number of effective homologous sequences aligned to residues, irrespective of residue type.

### SVM for microdeletions only

**Figure 2:**
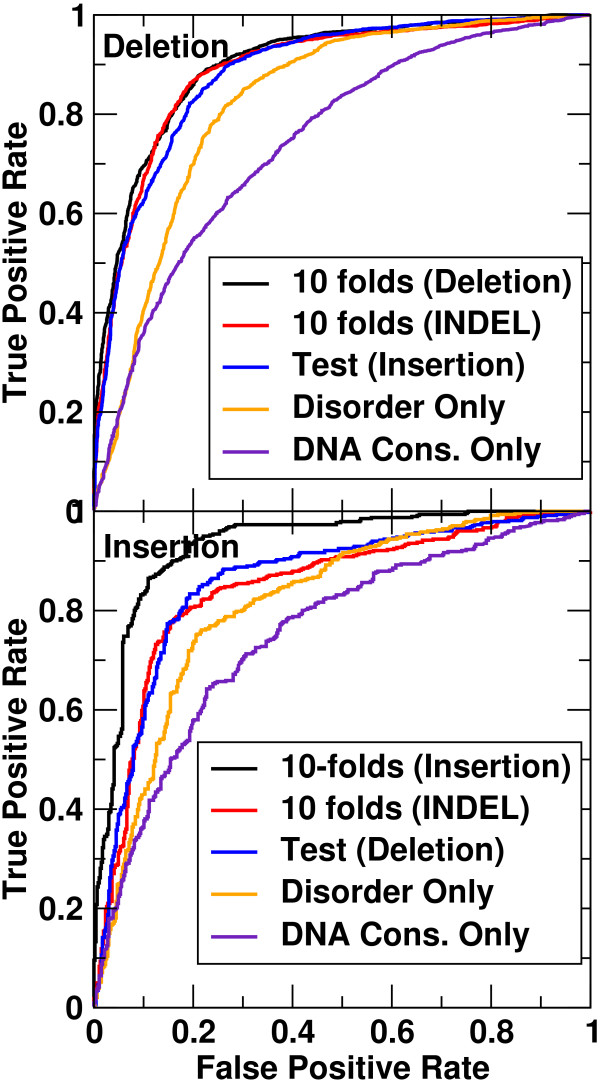
**The ROC curves for the microdeletion (top) and microinsertion (bottom) sets, respectively, by 10-fold cross-validation on the set (black), 10-fold cross-validation on both insertions and deletions (red), independent test by training on the microinsertions (top) or microdeletions (bottom) (blue), by disorder feature only (orange), and by DNA conservation score only (purple) as labeled**.

To combine different features for improving indel discrimination, we first employed support vector machines for the microdeletions. The microdeletion database included 1,998 disease-causing and 1,944 neutral NFS-indels. When all 58 features (listed in Table 1 and described in Methods) were employed, LIBSVM achieved an MCC value of 0.682, an accuracy of 84% and an AUC of 0.90 by 10-fold cross-validation. To avoid overtraining, and remove redundant features, we utilized a greedy feature selection method (see Methods) and selected 10 features as shown in Table 3. They were minimum disorder, maximum DNA conservation, microdeletion length, minimum ASA, average *HHBlits *match-to-microdeletion transition probability, the minimum effective number of aligned sequence to amino-acids, the distance to the nearest downstream splice site, maximum ASA, indel-induced change to matching score, and average ASA. The MCC and AUC values for this reduced feature set were 0.675 and 0.90, respectively. The precision and recall rates were 81% and 89%, respectively. The ROC curve from the 10-fold cross-validated result of the 10-feature model was compared to the results obtained from single features in Figure 2 (top panel). We tested the above models on the microinsertion dataset. We were able to treat the microinsertion dataset as a quasi-independent test set because only 21 proteins (from 743 proteins) harbored microinsertions and microdeletions at the same location. The full 58-feature model yielded an MCC value of 0.59, an accuracy of 74%, a precision of 82%, a recall of 76%, and an AUC of 0.84. By comparison, the above 10-feature model yielded an MCC value of 0.654, an accuracy of 83%, a precision of 82%, a recall of 85%, and an AUC of 0.86. This result is indicative of the same highly discriminating power of the microdeletion-trained model for microinsertions and highlights the importance of feature selection to avoid overtraining. Quantitatively similar behavior is observed in the precision-recall curve (Additional file 1, Figure S1).

**Table 3:** List of selected features for different training sets

Deletions	Insertions	indels	Non-redundant indels
Disorder(min)	Disorder(min)	Disorder(min)	Disorder(min)
DNA conservation(max)	DNA conservation(max)	DNA conservation(max)	DNA conservation(max)
Deletion length	P(m-i)^e ^(min)	ΔS^d^	ΔS^d^
ASA^a ^(min)	ΔS^d^	Neff^c^(ave)	Neff^c^(min)
P(m-d)^b^(ave)	P(m-i)^e ^(ave)	indel length	ASA^a ^(ave)
Neff^c^(min)	Disorder(ave)	Distance to the nearest splicing site (upstream)	indel length
Distance to the nearest splicing site (downstream)	Helical probability(max)	ASA^a ^(max)	ASA^a ^(max)
ASA^a^(max)	P(m-m)^f^(ave)	Neff^c^(min)	P(m-m)^f^(max)
ΔS^d^			DNA conservation(ave)
ASA(ave)			

^a^ASA, solvent accessible surface area. ^b^P(m-d), match-to-deletion transition probability. ^c^Neff: the number of effective homologous sequences aligned to residues. ^d^ΔS, indel-induced change to alignment score. ^e^P(m-i), match-to-insertion transition probability. ^f^P(m-m), match-to-match transition probability.

### SVM for microinsertions only

In a similar vein, we applied SVM to perform 10-fold cross-validation on the microinsertion set and employed the greedy feature selection to remove redundant features and avoid overtraining. This yielded a total of eight best performing features listed in Table 3. Three features (the minimum disorder probability, the DNA conservation, and indel-induced change to HMM match score) were the same as those in the 10-feature model for microdeletions. This eight-feature model achieved an MCC of 0.71, an accuracy of 86%, a precision of 85%, a recall of 86%, and an AUC of 0.88. This may be compared to 0.654 for MCC, 83% for accuracy, 82% for precision, 85% for recall, and 0.86 for AUC, the independent test result for the 10-feature model trained on the microdeletion dataset. The 10-fold cross-validation is more accurate than the independent test, in all probability due to the smaller size of the microinsertion dataset (only 481 and 446 disease-causing and putatively neutral microinsertions available for this analysis).

Application of this eight-feature model to the microdeletion dataset as an independent test set yielded an MCC of 0.64, an accuracy of 82%, a precision of 78%, a recall of 89%, and an AUC of 0.89. This result was comparable to 0.675 for MCC, 84% for accuracy, 81% for precision, 89% for recall, and 0.90 for AUC based on the10-fold cross-validation with 90% microdeletions as the training set for the 10-feature model. The ROC curve for microinsertions given by the eight-feature model (10-fold cross-validation) is compared to the ROC curves from single features of disorder and DNA conservation and the independent test result from the 10-feature model trained on microdeletions in Figure 2 (bottom panel). Essentially the same result is obtained in the precision-recall curve (Additional file 1, Figure S2).

### SVM for both microinsertions and microdeletions

The high discriminatory power of the microdeletion-trained model for microinsertions (and *vice versa*) suggested that it should be possible to treat microinsertions and microdeletions as a single dataset. The same feature selection procedure yielded a total of eight best-performing features for combined microinsertions and microdeletions as shown in Table 3. This set of features yielded 0.670 for MCC, 83% for accuracy and 0.89 for AUC. When we examined microdeletions and microinsertions separately, the results were 0.671 for the MCC, 84% for accuracy, and 0.89 for AUC in the case of microdeletions, 0.663 for the MCC, 83% for accuracy, and 0.88 for AUC in the case of microinsertions. The ROC curves given by the SVM model trained by both microinsertions and microdeletions yielded similarly accurate ROC curves given by independent tests for microdeletions or microinsertions, as shown in Figure 2. This further confirms the robustness of the SVM model.

### Effect of homologous sequences

The above results are based on datasets which had not had any homologous sequences removed. If a method is trained on one sequence and tested on a highly homologous sequence, the resulting accuracy estimate of the method may be inflated because of the similarity of the two sequences. The presence of homologous sequences may also bias training toward a particular type of protein. To explore such a possible effect, we reconstructed the SVM model based on the non-redundant set of NFS-indels (2,207 disease-causing and 2,241 neutral) in which all protein sequences exhibited ≤35% sequence identity between each other (see Methods). For this non-redundant set, the greedy-feature selection yielded nine best-performing features as shown in Table 3 and the final model with a 10-fold cross-validated MCC value of 0.684, accuracy of 84% precision of 81%, recall of 89%, and an AUC of 0.886. Application of this model back to the set without removing homologous sequences yielded an MCC of 0.71, an accuracy of 85%, precision of 81%, recall of 92%, and an AUC of 0.91. This result represented a marked improvement over 0.67 for MCC, 83% for accuracy, and 0.89 for AUC by training and cross-validating the same set. This confirms the importance of removing homologous proteins prior to training our SVM model. This SVM model is provided in Additional file 2.

### Minor allele frequency

**Figure 3:**
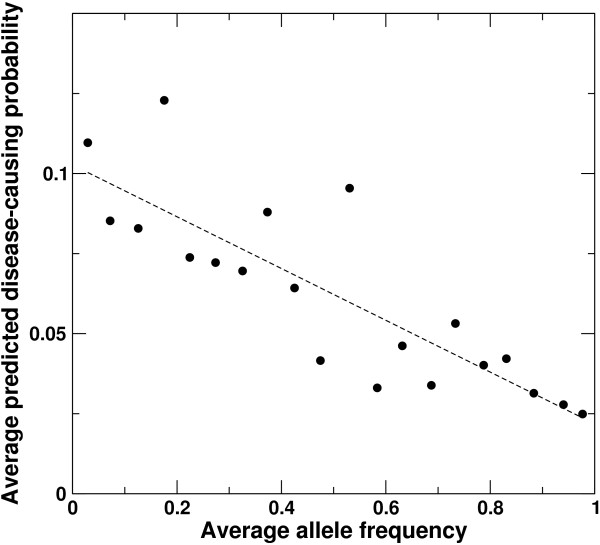
**The average predicted disease-causing probabilities as a function of the average allele frequency in the neutral indel dataset derived from the 1,000 Genomes Project data**. This was done by dividing allele frequencies into 20 bins. The dashed line is from a linear regression fit. The correlation coefficient is -0.84.

We obtained allele frequencies for all putatively neutral NFS-microdeletions and -microinsertions derived from the 1,000 Genomes Project data. The allele frequency in the population should in general reflect the fitness of that allele with respect to its intended biological function [20-24]. Figure 3 compares average predicted disease probabilities with average allele frequencies grouped into 20 bins (bin size, 0.05). The predicted disease probabilities are based on the 10-fold cross-validation by the nine-feature model trained on both microinsertions and microdeletions after removing homologous sequences. As expected, there was a strong negative correlation (correlation coefficient, -0.84), indicating that NFS-indels with higher predicted disease-causing probabilities tend to occur with lower allele frequencies in the general population.

## Discussion

We have developed a method, termed *DDIG-in*, for prioritizing NFS-indels by predicting the disease-causing probability for a given micro-indel. The method is based on nucleotide and amino-acid sequences and predicted structural features of proteins. The result suggests that highly accurate and robust prediction for both microinsertions and microdeletions can be made with only nine features. They are minimum disorder score, maximum DNA conservation score, the indel-induced change to the HMM alignment score, minimum effective number of aligned sequence to amino acids, average ASA, microinsertion/microdeletion length, maximum ASA, maximum *HHBlits *match-to-match transition probability, and average DNA conservation score. Interestingly, predicted ASA and DNA conservation are employed twice, once as the average value and a second time as the maximum value for the entire NFS-indel region. The difference between these two ASA or DNA conservation features measures the fluctuation of ASA or conservation for the indel region. The method was examined by 10-fold cross-validation as well as by an independent test. The consistency between 10-fold cross-validations and independent tests (84-85% for accuracy, 0.88-0.90 for AUC) supports the robustness of the final method developed.

One point to consider is that the most discriminating feature was predicted disordered (or structured) regions by SPINE-D. As Table 2 shows, the disorder feature alone can achieve an MCC value of 0.56 for both microinsertions and microdeletions. Although predicted disorder probabilities have previously been found to be useful in SNP discrimination [10,25], with disease-causing missense mutations being shown to be less likely to occur within disordered regions [26], its importance has never before been shown to be so prominent. This is probably due, at least in part, to the improvement of SPINE-D over previous algorithms [19]. It may also suggest the uniqueness of NFS-indel classification. This result is not unexpected because fully disordered regions (disorder probability, approximately 1) are structurally flexible and hence more permissive of modification by microinsertion or microdeletion as long as functional residues within the disordered regions remain intact. Indeed, we found that binding sites at intrinsically disordered regions of proteins are often located in semi-disordered regions (regions with a disorder probability of approximately 0.5) [27], consistent with near equal probability of disease-causing or neutral NFS-indels at disorder probability of approximately 0.5 in Figure 1.

Here, we assumed from the outset that the microdeletion and microinsertion variants identified during the course of the 1,000 Genomes Project are neutral. Although this assumption is not unreasonable, it should be appreciated that the training set may contain false negatives, especially for some late-onset disorders. To examine the effect of this, we removed those neutral variants with a minor allele frequency (MAF) of <2% and examined the effect of the removal of those variants on the accuracy and training of our NFS-indel discriminatory tool. This yielded 1,609 neutral cases plus 2,207 positive cases from the non-redundant set. The 10-fold cross-validation with the same nine features, but retrained without indels with a MAF of <2%, yielded an MCC of 0.70, an accuracy of 85%, and an AUC of 0.883. By comparison, application of the original nine-feature model (trained with neutral indels with a MAF of <2%) to the set of neutral indels without a MAF of <2% yielded an MCC of 0.74, an accuracy of 87%, and an AUC of 0.92. The fact that the nine-feature model trained without MAF <2% indels was less accurate than the nine-feature model trained with MAF <2% indels suggests that including MAF <2% indels (which potentially contained false negatives) facilitated machine learning. In other words, potential false negatives within the small frequency putatively neutral NFS-indels did not adversely affect SVM training. This is supported by strong negative correlations between the MAF and the disease-causing probability (Figure 3).

To further examine the effect of potential annotation errors in our datasets, we randomly introduced 5% or 10% errors to nine-folds by assigning neutral to disease-causing and disease-causing to neutral indels and testing the method for the remaining one fold. This was repeated 10 times. We also randomly introduced 5% or 10% errors 10 separate times to obtain an average effect. As described above, the 10-fold cross-validation with the same nine features (Table 3) but retrained without indels with a MAF of <2% yielded an MCC of 0.696. Adding 5% and 10% errors to nine training folds yielded the average MCC values for the test set of 0.684 and 0.674, respectively. This small change in MCC values due to 5% to 10% errors confirms that our method is robust against potential assignment errors in the training set.

**Figure 4:**
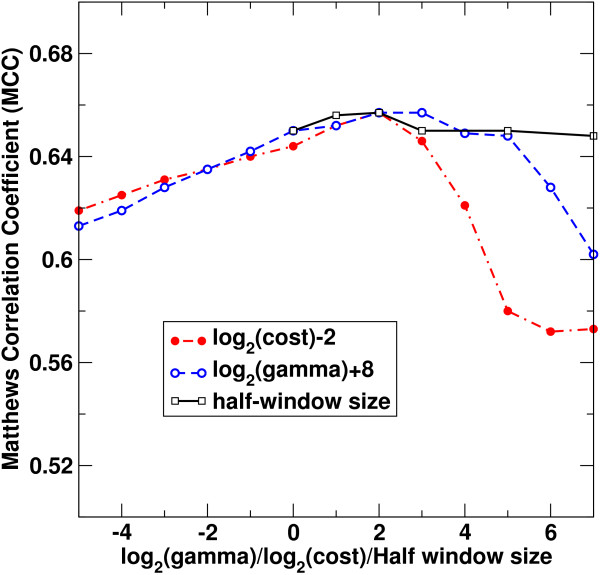
**Ten-fold cross-validated Matthews correlation coefficient for the NFS-indel set as a function of SVM gamma and cost parameters and half window size when trained on all features**. Note that a logarithmic scale is used for gamma and cost parameters and log_2_(gamma) and log_2_(cost) are shifted to facilitate comparison.

Another way to examine the robustness of a method is to test its dependence on various parameters. Figure 4 shows the Matthews correlation coefficient as a function of SVM gamma and cost parameters and the half-window size for the NFS-indel dataset for the case when all features were employed. It shows that MCC values change a little for the entire range of n_window _from 0 to 7 and for a large range of gamma and cost parameters.

Recently, Kumar *et al. *[28] found that most commonly used tools for non-synonymous SNV classification yield high false positive rates for ultra conserved sites. To examine the dependence of the accuracy of our method upon conservations of indel sites, we calculated conservation scores according to relative entropy (RE) [29]$\left[=100\sum_{\text{i = 1}}^{20}{\text{p}}_{\text{i}}\text{log}\left(\frac{{\text{p}}_{\text{i}}}{{\text{q}}_{\text{i}}}\right)\right]$ where ${\text{p}}_{\text{i}}$ is the probability of amino acid types at a sequence position obtained from PSI-BLAST [30], and ${\text{q}}_{\text{i}}$ is the background probability from the blosum62 matrix [31]. We divided our dataset into three portions (high, median, low) according to the average relative entropy of deleted residues or two residues around the insertion position (RE≥150, 70≤RE<150, RE<70). As in Kumar *et al*. [28], we also observed an elevated false positive rate at highly conserved sites (33%), relative to poorly conserved sites (14%). Interestingly, the true positive rate at highly conserved sites is also higher (95% at high RE sites *versus *72% at low RE sites). Thus, the overall performance of our method is not strongly dependent upon conservation of indel sites. The MCC values are 0.67, 0.63 and 0.58 for high, median and low RE indels, respectively. The relative independence of our method on the conservation of indel sites reflects the fact that sequence conservation is not the dominant feature in our indel discrimination technique. It should be noted that the threshold for indel discrimination can be modified for high RE sites to reduce the somewhat elevated false positive rate.

It is worthy of note that the indel length is one of the top features selected by SVM. This is reasonable because longer indels will likely have greater impact upon protein structure and function. However, it could also be due to bias in our datasets because, empirically, the majority of indels involve short lengths of one or two residues in both our datasets, a reflection of the inherent bias of the underlying mutational mechanism *in vivo*. Such an unbalanced dataset renders size-controlled or stratified sampling impossible. Thus, to determine whether the length dependence is a result of dataset bias or is instead of true functional origin would require further studies employing much larger datasets for both disease-causing and neutral indels. Nevertheless, the effect of this feature on the overall accuracy is small. Removing this feature only decreases the MCC value from 0.684 to 0.664 for our non-redundant indel sets.

In addition to the features listed in Table 1 we also performed a test for the usefulness of biochemical properties of amino acid residues such as residue size and hydrophobicity scale for indel discrimination. This is in part because such features have been found to be effective in protein secondary structure prediction [32,33]. We examined seven representative physical parameters including a steric parameter (graph shape index), hydrophobicity, volume, polarizability, isoelectric point, helix probability, and sheet probability [32,33]. None of these features were found to further improve the MCC value for indel discrimination.

This work is consistent with various studies that have examined the sequence context of microdeletions and microinsertions. These studies found that indels occurred non-randomly and were highly influenced by the local DNA sequence context [2,34,35]. This probably accounts for the success of our algorithm in NFS-indel classification based upon local sequence and structural information. Furthermore, microinsertions and microdeletions exhibit strong similarities in terms of the characteristics of their flanking DNA sequences, implying that they are generated by very similar underlying mechanisms [2]. Again, this accords with our ability to design a single tool capable of discriminating between microdeletions and microinsertions of pathological importance and neutral microdeletions/microinsertions.

This study focused on NFS-indels only because FS-indels would require a quite separate algorithm to effect their classification. Such an algorithm would require features based on the entire region after the indel site, rather than simply the local region around the indel site. This is because the frame-shift in FS-indels results either in a completely different amino-acid sequence C-terminal to the indel site or premature termination of translation. Expansion of *DDIG-in *so as to include FS-indels is however in progress. In the meantime, our sequence- and structure-based tool will complement two recently developed methods [16,17] that are based on information derived only from nucleotide and amino-acid sequences. In addition to extension to cover FS-indels, we intend to incorporate new features other than sequence- and structure-based features. Other such features (for example, predicted functional regions) may well be useful in further improving the micro-indel classification as was previously achieved for SNP classification [11-14].

## Materials and methods

### Dataset of positive indels

The positive (disease-causing) dataset was obtained from the HGMD (HGMD Professional v. 2012.2) [1]. Initially, a total of 25,384 indels were identified after mapped to CCDS (20110907 version). After excluding frameshift (FS) indels and those indels that were located in an intron or at a stop codon, we obtained a dataset of 2,479 exonic disease-causing NFS-indels in 743 protein-coding genes. Of these, 1,998 and 481 were microdeletions and microinsertions, respectively. To examine the possible effect of homologous sequences on training our bioinformatics method, we also constructed a non-redundant dataset lacking homologous sequences that had >35% sequence identity between any pair of sequences. This was accomplished by pairwise sequence alignment and clustering by BlastClust [30] and only one representative sequence was chosen from each cluster. A 35% protein sequence identity cutoff was employed because this cutoff lies at the boundary that distinguishes close homologs from remote homologs [36,37]. This removal of homologous sequences yielded 1,762 microdeletions and 445 microinsertions from 680 protein-coding genes. We also examined the overlap between microinsertion and microdeletion datasets. We considered that a microinsertion and a microdeletion were located at the same site if at least one of the two nearest neighboring residues flanking the inserted residues in the microinsertion contributed to the deleted residues in the microdeletion. This definition yielded 21 of 743 proteins; they were CCDS13330.1, CCDS8539.1, CCDS13989.1, CCDS5313.1, CCDS2145.1, CCDS30981.1, CCDS747.1, CCDS4306.1, CCDS13858.1, CCDS5773.1, CCDS6392.1, CCDS1390.1, CCDS11892.1, CCDS14083.1, CCDS10458.1, CCDS12198.1, CCDS2463.1, CCDS11453.1, CCDS11127.1, CCDS1071.1, and CCDS45080.1. The minimal overlap suggested that the microinsertion and microdeletion sets could to all intents and purposes be treated as independent test datasets against each other.

### Dataset of putatively neutral indels

The putatively neutral dataset was retrieved from the micro-indel variants identified during the 1,000 Genomes Project (20101123 release), in which apparently healthy individuals from five major populations were sequenced [38]. As with the HGMD data, the indels were located using hg19 as the reference genome. From 9,327 exonic indels (excluding more than 3 million intronic indels), we identified a total of 2,413 NFS-indels of which 1,944 were microdeletions and 469 were microinsertions. These 2,413 NFS-indels were derived from 1,929 protein-coding genes after excluding FS-indels and those indels that were located in an intron or at a stop codon. Removal of homologous sequences (based on a protein sequence identity cut-off of 35%), yielded 1,795 microdeletions and 446 microinsertions (a total of 2241 neutral micro-indels) from 640 protein-coding genes. Unlike the disease-causing NFS-indel dataset, there was no overlap between the positions of the microdeletions and those of the microinsertions in this dataset. Minor allele frequencies were retrieved for all 2,241 NFS-indels from the 1,000 Genomes Project. Both datasets (with and without homologous sequences) were employed to train and test our models to examine the effect of homologous sequences. It should be noted however that we cannot wholly exclude the possibility that a small subset of this putatively neutral dataset could still be of functional importance (more in the Discussion section).

### Structural and sequence features

We tested many features for their potential roles in indel discrimination. These features are summarized in Table 1 and are described in detail below.

#### Nucleotide sequence-level features

We examined the following nucleotide sequence-level features as potential discriminators between disease-causing and neutral NFS-indels: the distances from the indel site to the nearest upstream and downstream splice sites and the DNA conservation score derived from phyloP (phylogenetic *P *values) [39]. We examined the distances from nearest splice sites because mutations near splice sites have the potential to give rise to alternative splicing patterns [40]. All DNA conservation scores downloaded from [41]were based on multiple alignments of 45 vertebrate genomes to the human genome. To calculate a DNA conservation score for a microdeletion, we considered all the deleted bases (n_del_) plus a fixed number of bases before and after the deleted bases (the half-window size, n_window_). We obtained the average, minimum, and maximum DNA conservation scores based on phylogenetic *P *values over the specified bases around the deleted bases (that is, n_del_+2n_window_). For microinsertions, we considered the two bases flanking the microinsertion plus a fixed number of additional neighboring upstream and downstream bases (that is, 2+2n_window_). The maximum, minimum and average conservation scores for 2+2n_window _bases were also obtained. These five nucleotide sequence-level features (2 distances+3×1 DNA conservation scores) were studied here to assess their utility in indel classification.

#### Protein sequence-level features

We obtained features at the amino-acid sequence level using a program called *HHBlits *that derives multiple protein sequence alignments based on profiles generated from hidden Markov chain models (HMM) [42] (downloaded from [43]).This program compares two sequences at the HMM profile level and searches for homologous sequences from the UniProt sequence database. It is a more sensitive technique than the sequence-to-profile homolog search tool *PSI-BLAST *[30] commonly used in classifications of non-synonymous SNVs (for example, SIFT [8]) because *HHBlits *employs a position-dependent gap penalty and calculates transition probabilities not only between matches of two residues (that is, two residues from two sequences are aligned) but also between other states (match to microdeletion, match to microinsertion, microdeletion to match, microinsertion to match, microinsertion to microinsertion, and microdeletion to microdeletion). That is, there are a total of seven position-dependent transition probabilities. In addition, for each position, we can obtain three effective numbers of homologous sequences (n_eff_) aligned to microinsertion, to microdeletion and to amino-acid residues, irrespective of residue type. The maximum, minimum, and average of all these amino-acid residue level properties (3×(7+3)=30 features) were obtained for a specified region. For the microdeletions, this region included deleted residues plus several residues before and after the deleted residues (n_del_+2n_window_). For microinsertions, this region comprised the two nearest neighboring residues flanking the inserted residues plus a fixed number of residues before and after these two residues (2+2n_window_). In addition, we calculated a global protein feature: the change to the HMM-HMM alignment score by the whole protein sequence before and after the microdeletion or microinsertion. We also examined four features of microinsertion/microdeletion length, protein length and distances to the protein amino and carboxyl terminal ends. A total of 35 features (30+1+4) were generated from protein sequences.

#### Protein structure-level features

The first protein structure-level feature was based on amino acid sequence-based prediction of structured and unstructured regions by a neural-network-based disorder predictor, SPINE-D [19]. We employed SPINE-D because it is among the most accurate methods based on benchmarks [19] according to the 9th Meeting for Critical Assessment of Structure Prediction Techniques (CASP 9, 2010) [19,44]. We examined the maximum, minimum and average values of disorder probabilities over the specified region described above (n_del_+2n_window _for microdeletion_, _2+2n_window _for microinsertion). In addition, we obtained predicted secondary structures, secondary structure probability, and solvent accessible surface area for the same specified region from SPINE-X [33]. SPINE-X has achieved 82% accuracy in secondary structure prediction [33] and 0.74 for the correlation coefficient between predicted and measured solvent accessible surface area (ASA) [45] based on large-scale benchmark tests. As with the disorder feature, we obtained the maximum, minimum, and average values of predicted secondary probabilities in three states and predicted real-value solvent accessibility over the specified region for microdeletions or microinsertions. We also studied the fractions of three secondary structure types over the same specified region. A total of 18 structure-based features (3×1 disorder, 3 fractions of secondary structure types, 3×3 secondary structure probability, and 3×1 ASA) were generated for studies.

### Parameter optimization for SVM

We employed LIBSVM (LIBSVM: a library for support vector machines (SVM)) [46] to combine the features listed above for NFS-indel classification. There are two parameters for SVM: a non-linear kernel of radial basis function with a gamma parameter and the cost parameter (C) that allows a soft region for misclassification. In addition, we employed a half-window size (n_window_) to include several amino-acid residues before and after the microdeletion/microinsertion site as defined above. For example, a half-window size of 0 would contain all residues deleted in a microdeletion and two residues flanking the inserted residues for a microinsertion. To reduce the number of parameters, a uniform widow size was applied to all features requiring a window size. A simple grid search was done with a grid of 2 ranging from -5 to 15 for logC and ranging -15 to 3 for log(gamma), and a window size ranging from 0 to 7. That is, we searched for the parameters that yielded the highest Matthews correlation coefficient (MCC) for 10-fold cross-validations (nine-fold for training and one- fold for testing) while employing all features. We also examined the dependence of MCC values on C, gamma, and n_window _and found that MCC values change little across a wide range of C, gamma, and n_window _values (see Discussion). This served to confirm the robustness of the parameters we found.

### Evaluation of overall performance

The overall performance of NFS-indel classifications was assessed by the ROC curve and area under the ROC curve (AUC) for training and test sets. In addition, we also calculated recall (also called sensitivity) (*TP*/(*TP+FN*)), precision (*TP*/(*TP+FP*)), accuracy (*ACC= *(*TP+TN*)/(*TP+FP+FN+TN*)), and Matthews correlation coefficient $MCC=\left(TP\times TN-FP\times FN\right)/\sqrt{\left(TP+FP\right)\left(TP+FN\right)\left(TN+FP\right)\left(TN+FN\right)}$ where *TP*, *FP*, *TN*, and *FN *denote true positives (correctly predicted disease-causing NFS-indels), false positives (neutral NFS-indels predicted to be disease-causing), true negatives (correctly predicted neutral NFS-indels), and false negatives (disease-causing NFS-indels predicted to be neutral), respectively. The Matthews correlation coefficient, 1 for perfect prediction and 0 for random prediction, is a balanced measure of true/false positives and negatives [44]. It served as the key parameter for performance measurement.

### Training and cross-validation

The training set (positive and putatively neutral datasets) was randomly divided into 10 parts, nine of which were used for training, the rest for testing. This process was repeated 10 times (10-fold cross-validation). We performed 10-fold cross-validation on SVM models for microdeletions or microinsertions only, as well as for the combined set of microdeletions and microinsertions. Microinsertion and microdeletion datasets were also used as independent test sets against each other in order to evaluate the overall robustness of the classification technique employed. In other words, the methods trained with the microinsertion set never 'saw' the microdeletion dataset and *vice versa*.

### Feature selections

To identify the most informative subset of features, a previously described greedy feature selection algorithm for SNV classification [47] was employed. This iterative greedy algorithm starts with the feature shown to have the highest discriminatory power (disease *versus *neutral) based on the MCC value. The second feature was then selected on the basis that the combination of the first and the second features yielded the highest MCC value among all combinations between the first and other features. Similarly, the third feature was added to the first two if the addition of the third feature further improved MCC and the improvement was the largest obtained by comparison with the other remaining features. The iteration of adding an additional feature from the remaining features was halted if the MCC value failed to increase. Here, the MCC value was derived from the 10-fold cross-validation.

## List of abbreviations

ASA: Solvent accessible surface area; AUC: Area under the curve; CASP: Critical assessment of structure prediction techniques; CCDS: Consensus coding sequence; DDIG-in: Detecting disease-causing genetic variations due to indels; FS: Frameshifting; HGMD: Human genome mutation database; HHblits: HMM-HMM-based lightning-fast iterative sequence search; HMM: Hidden Markov model; LIBSVM: Library for support vector machines; MAF: Minor allele frequency; MCC: Matthews correlation coefficient; MutPred: Mutation prediction; NFS: Non-frameshifting; PolyPhen: Polymorphism Phenotyping; PSI-BLAST: Position-specific iterated BLAST; RE: Relative entropy; ROC curve: Receiver-operating characteristic curve; SIFT: Sorts intolerant from tolerant amino acid substitutions; SNV: Single nucleotide variant; SPINE-D: Sequence-based prediction with integrated neural networks for protein disorder; SVM: Support vector machines; UniProt: the universal protein resource.

## Competing interests

The authors declare that they have no competing interests.

## Authors' contributions

HZ and YY integrated the data for training and testing the DDIG-in method. HL and XZ obtained and compiled data from the 1,000 Genomes Project. MM and DNC obtained and compiled the HGMD data. HZ, YY, YL, and YZ designed the studies. HZ, YY, DNC, YL, and YZ wrote the paper. All authors have read and approved this manuscript for publication.

## Supplementary Material

Additional file 1 **Tables S1 and S2; Figures S1 and S2**. Click here for file

Additional file 2 **The text file for the SVM model**. Click here for file
