# Supplementary material for: DDIG-in: discriminating between disease-associated and neutral non-frameshifting micro-indels
Source: Genome Biol. 2013 Mar 13;14(3):R23. doi: 10.1186/gb-2013-14-3-r23 (PMC4053752; doi:10.1186/gb-2013-14-3-r23)
Supplement: Additional file 1 — Tables S1 and S2; Figures S1 and S2. [file gb-2013-14-3-r23-S1.DOCX]

**Supplemental Material for “Discriminating between disease-causing and neutral non-frameshifting micro-INDELs by support vector machines by means of integrated sequence- and structure-based features”**

Huiying Zhao^1,§^, Yuedong Yang^1,2,§^, Hai Lin^2^, Xinjun Zhang^2^, Matthew Mort^4^, David N. Cooper^4^, Yunlong Liu^2,3,*^, and Yaoqi Zhou^1,2,^*

^1^School of Informatics, Indiana University Purdue University, Indianapolis, Indiana 46202, USA

^2^Center for Computational Biology and Bioinformatics, Indiana University School of Medicine, Indianapolis, Indiana 46202, USA

^3^Department of Medical and Molecular Genetics, Indiana University School of Medicine, Indianapolis, Indiana 46202, USA

^4^Institute of Medical Genetics, Cardiff University, Heath Park, Cardiff CF14 4XN, UK

^§^Equal contribution.

* To whom correspondence should be addressed. Dr. Liu (Tel. 317-278-9222; Fax. 317-278-9217;

[yunliu@iupui.edu](mailto:yunliu@iupui.edu)); Dr. Zhou (Tel: 317-278-7674; Fax: 317-278-9201; Email: yqzhou@iupui.edu)

| Table S1. Performance of individual features (Deletion) | | | | | | | |
| --- | --- | --- | --- | --- | --- | --- | --- |
| Features | | | | | MCC^a^ | AUC^b^ | ACC^c^ |
| Disorder | | | | Max | 0.551 | 0.818 | 0.772 |
|  |  |  |  | Min | 0.558 | 0.824 | 0.777 |
|  |  |  |  | Average | 0.557 | 0.825 | 0.777 |
| SS^d^ | | | | C | 0.235 | 0.634 | 0.613 |
|  |  |  |  | H | 0.131 | 0.595 | 0.562 |
|  |  |  |  | E | 0.218 | 0.616 | 0.578 |
| SS^d^ Probability and ASA | | Max | | C | 0.258 | 0.655 | 0.627 |
|  |  |  |  | H | 0.218 | 0.613 | 0.6 |
|  |  |  |  | E | 0.32 | 0.678 | 0.658 |
|  |  |  |  | ASA^e^ | 0.302 | 0.659 | 0.648 |
|  |  | Min | | C | 0.185 | 0.6 | 0.585 |
|  |  |  |  | H | 0.263 | 0.671 | 0.628 |
|  |  |  |  | E | 0.305 | 0.658 | 0.647 |
|  |  |  |  | ASA^e^ | 0.542 | 0.81 | 0.766 |
|  |  | Aver | | C | 0.256 | 0.658 | 0.624 |
|  |  |  |  | H | 0.223 | 0.627 | 0.605 |
|  |  |  |  | E | 0.284 | 0.632 | 0.635 |
|  |  |  |  | ASA | 0.47 | 0.781 | 0.733 |
| Evo^f^ | Aver | | | M-M | 0.234 | 0.64 | 0.617 |
|  |  |  |  | M-I | 0.263 | 0.655 | 0.63 |
|  |  |  |  | M-D | 0.155 | 0.584 | 0.577 |
|  |  |  |  | I-M | 0.258 | 0.654 | 0.629 |
|  |  |  |  | I-I | 0.256 | 0.657 | 0.628 |
|  |  |  |  | D-M | 0.181 | 0.596 | 0.591 |
|  |  |  |  | D-D | 0.182 | 0.605 | 0.587 |
|  |  |  |  | Neff^g^ | 0.439 | 0.749 | 0.711 |
|  |  |  |  | Neff_I | 0.259 | 0.668 | 0.626 |
|  |  |  |  | Neff_D | 0.162 | 0.591 | 0.574 |
|  | Min | | | M-M | 0.176 | 0.604 | 0.573 |
|  |  |  |  | M-I | 0.308 | 0.676 | 0.653 |
|  |  |  |  | M-D | 0.284 | 0.671 | 0.639 |
|  |  |  |  | I-M | 0.261 | 0.643 | 0.625 |
|  |  |  |  | I-I | 0.234 | 0.604 | 0.615 |
|  |  |  |  | D-M | 0.163 | 0.577 | 0.578 |
|  |  |  |  | D-D | 0.113 | 0.567 | 0.551 |
|  |  |  |  | Neff^g^ | 0.449 | 0.735 | 0.719 |
|  |  |  |  | Neff_I | 0.124 | 0.58 | 0.537 |
|  |  |  |  | Neff_D | 0.132 | 0.581 | 0.562 |
|  | Max | | | M-M | 0.292 | 0.651 | 0.644 |
|  |  |  |  | M-I | 0.136 | 0.58 | 0.54 |
|  |  |  |  | M-D | 0.116 | 0.571 | 0.526 |
|  |  |  |  | I-M | 0.127 | 0.571 | 0.537 |
|  |  |  |  | I-I | 0.0925 | 0.564 | 0.52 |
|  |  |  |  | D-M | 0.0882 | 0.572 | 0.523 |
|  |  |  |  | D-D | 0.145 | 0.579 | 0.568 |
|  |  |  |  | Neff^g^ | 0.43 | 0.729 | 0.708 |
|  |  |  |  | Neff_I | 0.287 | 0.709 | 0.641 |
|  |  |  |  | Neff_D | 0.219 | 0.621 | 0.60 |
| DNA Conser. | | | Aver | | 0.367 | 0.742 | 0.683 |
|  |  |  | Max | | 0.468 | 0.781 | 0.733 |
|  |  |  | Min | | 0.144 | 0.561 | 0.557 |
| Deletion length | | | INDEL len | | 0.263 | 0.651 | 0.617 |
|  |  |  | Protein Len | | 0.134 | 0.573 | 0.567 |
|  |  |  | Dis to head | | 0.121 | 0.564 | 0.555 |
|  |  |  | Dis to tail | | 0.103 | 0.542 | 0.55 |
| Splicing position | | | To head | | 0.219 | 0.606 | 0.596 |
|  |  |  | To tail | | 0.24 | 0.631 | 0.612 |
| ΔS^h^ | | |  | | 0.285 | 0.676 | 0.633 |

^a^MCC: Mathews correlation coefficient. ^b^AUC: area under the curve. ^c^ACC^:^ Accuracy. ^d^SS: predicted secondary structure. ^e^ASA, solvent accessible surface area. ^f^Evo: Evolutionary information generated by HHblits. ^g^Neff: the number of effective homologous sequences aligned to residues, irrespective of residue type. ^h^ΔS: the INDEL-induced change to the HMM match score.

| Table S2. Performance of individual features (Insertion) | | | | | | | |
| --- | --- | --- | --- | --- | --- | --- | --- |
| Features | | | | | MCC^a^ | AUC^b^ | ACC^c^ |
| Disorder | | | | Max | 0.546 | 0.816 | 0.772 |
|  |  |  |  | Min | 0.556 | 0.813 | 0.777 |
|  |  |  |  | Average | 0.545 | 0.80 | 0.772 |
| SS^d^ | | | | C | 0.321 | 0.674 | 0.657 |
|  |  |  |  | H | 0.146 | 0.584 | 0.565 |
|  |  |  |  | E | 0.25 | 0.817 | 0.589 |
| SS^d^ Probability and ASA | | Max | | C | 0.314 | 0.688 | 0.657 |
|  |  |  |  | H | 0.254 | 0.646 | 0.627 |
|  |  |  |  | E | 0.349 | 0.698 | 0.674 |
|  |  |  |  | ASA^e^ | 0.317 | 0.67 | 0.652 |
|  |  | Min | | C | 0.224 | 0.621 | 0.609 |
|  |  |  |  | H | 0.317 | 0.694 | 0.652 |
|  |  |  |  | E | 0.346 | 0.663 | 0.669 |
|  |  |  |  | ASA^e^ | 0.501 | 0.80 | 0.605 |
|  |  | Aver | | C | 0.312 | 0.692 | 0.656 |
|  |  |  |  | H | 0.328 | 0.695 | 0.66 |
|  |  |  |  | E | 0.306 | 0.636 | 0.646 |
|  |  |  |  | ASA^e^ | 0.454 | 0.78 | 0.724 |
| Evo^f^ | Aver | | | M-M | 0.246 | 0.628 | 0.623 |
|  |  |  |  | M-I | 0.182 | 0.614 | 0.588 |
|  |  |  |  | M-D | 0.186 | 0.597 | 0.589 |
|  |  |  |  | I-M | 0.199 | 0.619 | 0.588 |
|  |  |  |  | I-I | 0.197 | 0.609 | 0.597 |
|  |  |  |  | D-M | 0.208 | 0.616 | 0.602 |
|  |  |  |  | D-D | 0.171 | 0.544 | 0.542 |
|  |  |  |  | Neff^g^ | 0.455 | 0.747 | 0.72 |
|  |  |  |  | Neff_I | 0.226 | 0.619 | 0.603 |
|  |  |  |  | Neff_D | 0.178 | 0.566 | 0.579 |
|  | Min | | | M-M | 0.15 | 0.581 | 0.544 |
|  |  |  |  | M-I | 0.372 | 0.708 | 0.684 |
|  |  |  |  | M-D | 0.326 | 0.667 | 0.656 |
|  |  |  |  | I-M | 0.199 | 0.586 | 0.595 |
|  |  |  |  | I-I | 0.16 | 0.58 | 0.574 |
|  |  |  |  | D-M | 0.158 | 0.589 | 0.574 |
|  |  |  |  | D-D | 0.0965 | 0.493 | 0.509 |
|  |  |  |  | Neff^g^ | 0.467 | 0.751 | 0.727 |
|  |  |  |  | Neff_I | 0.0782 | 0.545 | 0.508 |
|  |  |  |  | Neff_D | 0.107 | 0.508 | 0.528 |
|  | Max | | | M-M | 0.337 | 0.681 | 0.66 |
|  |  |  |  | M-I | 0.123 | 0.546 | 0.513 |
|  |  |  |  | M-D | 0.0926 | 0.548 | 0.509 |
|  |  |  |  | I-M | 0.105 | 0.54 | 0.508 |
|  |  |  |  | I-I | 0.136 | 0.572 | 0.519 |
|  |  |  |  | D-M | 0.109 | 0.55 | 0.517 |
|  |  |  |  | D-D | 0.152 | 0.568 | 0.551 |
|  |  |  |  | Neff^g^ | 0.438 | 0.742 | 0.713 |
|  |  |  |  | Neff_I | 0.232 | 0.625 | 0.614 |
|  |  |  |  | Neff_D | 0.23 | 0.601 | 0.612 |
| DNA Conser. | | | Aver | | 0.422 | 0.752 | 0.709 |
|  |  |  | Max | | 0.453 | 0.758 | 0.727 |
|  |  |  | Min | | 0.234 | 0.597 | 0.585 |
| Deletion length | | | INDEL len | | 0.02 | 0.9 | 0.507 |
|  |  |  | Protein Len | | 0.116 | 0.532 | 0.555 |
|  |  |  | Dis to head | | 0.172 | 0.57 | 0.58 |
|  |  |  | Dis to tail | | 0.121 | 0.553 | 0.561 |
| Splicing position | | | To head | | 0.186 | 0.576 | 0.576 |
|  |  |  | To tail | | 0.283 | 0.651 | 0.64 |
| ΔS^h^ | | |  | | 0.303 | 0.673 | 0.629 |

^a^MCC: Mathews correlation coefficient. ^b^AUC: area under the curve. ^c^ACC^:^ Accuracy. ^d^SS: predicted secondary structure. ^e^ASA, solvent accessible surface area. ^f^Evo: Evolutionary information generated by HHblits. ^g^Neff: the number of effective homologous sequences aligned to residues, irrespective of residue type. ^h^ΔS: the INDEL-induced change to the HMM match score.

Figure S1.


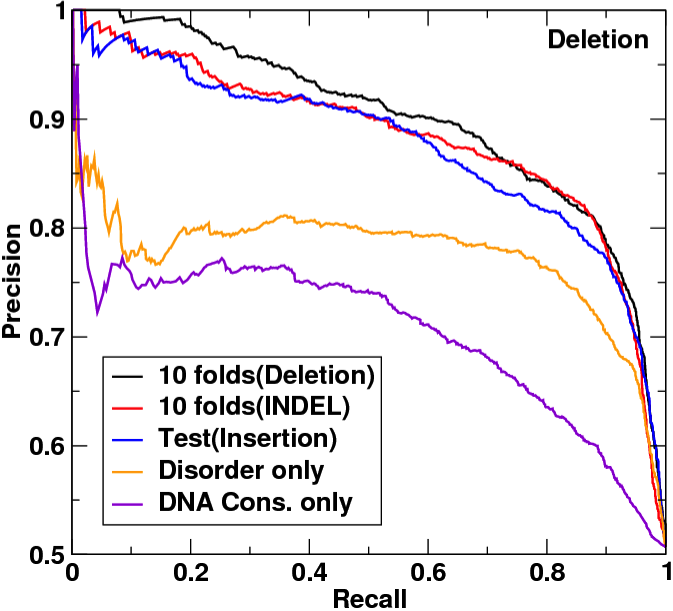


Figure S1 Precision versus recall curve for the microdeletion dataset by ten-fold cross-validation on the deletion set (black), ten-fold cross-validation on both insertions and deletions (Red), independent test by training on the microinsertions (Blue), by disorder feature only (Orange) and by DNA conservation score only (Purple) as labeled.

Figure S2


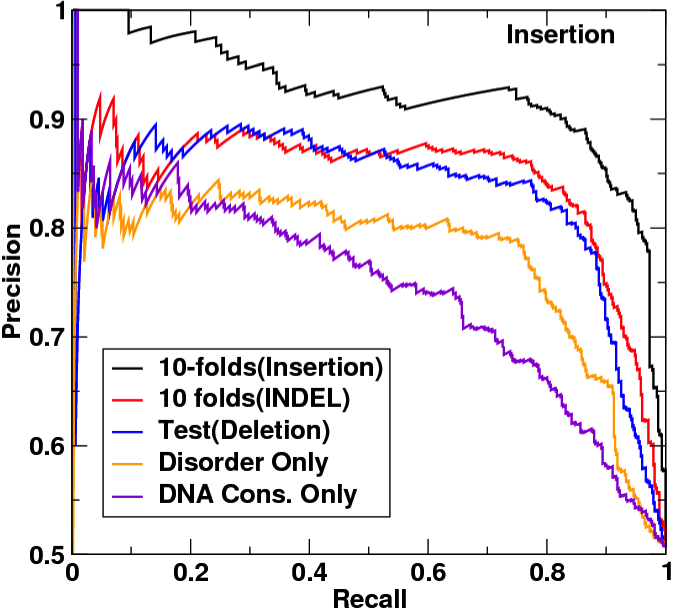


Figure S2 Precision versus recall curve for the microinsertion dataset by ten-fold cross-validation on the insertion set (black), ten-fold cross-validation on both insertions and deletions (Red), independent test by training on microdeletions (Blue), by disorder feature only (Orange) and by DNA conservation score only (Purple) as labeled.
